# Proteoliposomal formulations of an HIV-1 gp41-based miniprotein elicit a lipid-dependent immunodominant response overlapping the 2F5 binding motif

**DOI:** 10.1038/srep40800

**Published:** 2017-01-13

**Authors:** Luis M. Molinos-Albert, Eneritz Bilbao, Luis Agulló, Silvia Marfil, Elisabet García, Maria Luisa Rodríguez de la Concepción, Nuria Izquierdo-Useros, Cristina Vilaplana, Jon A. Nieto-Garai, F.-Xabier Contreras, Martin Floor, Pere J. Cardona, Javier Martinez-Picado, Bonaventura Clotet, Jordi Villà-Freixa, Maier Lorizate, Jorge Carrillo, Julià Blanco

**Affiliations:** 1 AIDS Research Institute IrsiCaixa-HIVACAT, Institut de Recerca en Ciències de la Salut Germans Trias i Pujol (IGTP), Hospital Germans Trias i Pujol, Universitat Autònoma de Barcelona, 08916 Badalona, Barcelona, Catalonia, Spain; 2 Biofisika Institute (UPV/EHU, CSIC) and Department of Biochemistry and Molecular Biology, Universidad del País Vasco (UPV/EHU), 48940, Bilbao, Spain; 3 Department of Biosciences, U Science Tech (UST), Universitat de Vic - Universitat Central de Catalunya (UVic-UCC), 08500 Vic, Barcelona, Spain; 4 Unitat de Tuberculosi Experimental, Institut de Recerca en Ciències de la Salut Germans Trias i Pujol (IGTP), 08916 Badalona, Barcelona, Catalonia, Spain; 5 Ikerbasque Basque Foundation for Science, 48011 Bilbao, Spain; 6 Molecular Graphics Suite, Department of Inorganic and Analytical Chemistry, Faculty of Chemical and Pharmaceutical Sciences, University of Chile, Santiago, Chile; 7 Catalan Institution for Research and Advanced Studies (ICREA), Barcelona, Spain; 8 Chair on AIDS and related diseases, Universitat de Vic - Universitat Central de Catalunya (UVic-UCC), 08500 Vic, Barcelona, Spain; 9 Fundació Lluita contra la Sida, Hospital Germans Trias i Pujol, 08916 Badalona, Barcelona, Catalonia, Spain

## Abstract

The HIV-1 gp41 Membrane Proximal External Region (MPER) is recognized by broadly neutralizing antibodies and represents a promising vaccine target. However, MPER immunogenicity and antibody activity are influenced by membrane lipids. To evaluate lipid modulation of MPER immunogenicity, we generated a 1-Palmitoyl-2-oleoylphosphatidylcholine (POPC)-based proteoliposome collection containing combinations of phosphatidylserine (PS), GM3 ganglioside, cholesterol (CHOL), sphingomyelin (SM) and the TLR4 agonist monophosphoryl lipid A (MPLA). A recombinant gp41-derived miniprotein (gp41-MinTT) exposing the MPER and a tetanus toxoid (TT) peptide that favors MHC-II presentation, was successfully incorporated into lipid mixtures (>85%). Immunization of mice with soluble gp41-MinTT exclusively induced responses against the TT peptide, while POPC proteoliposomes generated potent anti-gp41 IgG responses using lower protein doses. The combined addition of PS and GM3 or CHOL/SM to POPC liposomes greatly increased gp41 immunogenicity, which was further enhanced by the addition of MPLA. Responses generated by all proteoliposomes targeted the N-terminal moiety of MPER overlapping the 2F5 neutralizing epitope. Our data show that lipids impact both, the epitope targeted and the magnitude of the response to membrane-dependent antigens, helping to improve MPER-based lipid carriers. Moreover, the identification of immunodominant epitopes allows for the redesign of immunogens targeting MPER neutralizing determinants.

The HIV-1 envelope glycoprotein (Env) is a trimer of heterodimers composed by the non-covalent association of gp120 and gp41 subunits^1^. It is the sole viral protein exposed on the viral surface and, thus, is the main target of neutralizing antibodies. In spite of more than 30 years of research, an immunogen capable of inducing a broadly neutralizing antibody response against Env has not been achieved yet. Due to the high viral variation rate and immune evasion, a successful preventive vaccine should target conserved functional epitopes within the envelope. The identification of a small percentage of broadly neutralizing humoral responses in different cohorts of HIV-1 infected individuals highlighted the uncommonness but also the feasibility to develop this kind of responses by the human immune system^2^,^3^,^4^,^5^,^6^. Furthermore, the isolation of broadly neutralizing antibodies (bNAbs) from these individuals identified several antigenic vulnerability sites within Env including the CD4 binding site^4^,^7^,^8^,^9^,^10^; glycan-dependent epitopes defined by residues N160 and N332 in the V1/V2 and V3 gp120 loops respectively^10^,^11^,^12^; the gp41 Membrane Proximal External Region (MPER)^13^,^14^,^15^,^16^; and recently discovered regions including residues from both gp120 and gp41^17^,^18^,^19^ and the gp41 fusion peptide^20^. The study of these regions has guided efforts in HIV-1 vaccine development during the last years^21^,^22^.

The MPER is a highly conserved tryptophan-rich region that has crucial roles in both viral fusion^23^,^24^ and CD4-independent transcytosis across the epithelial cell barriers^25^. Moreover, the MPER includes linear, transiently exposed epitopes targeted by bNAbs such as 2F5, 4E10 and 10E8. All of them show a wide *in vitro* activity and are able to protect animals upon viral challenge *in vivo*^16^,^26^,^27^,^28^. Particularly 10E8 is one of the broadest and most potent bNAbs isolated to date^16^. Therefore, the MPER is considered as a potential HIV-1 vaccine target. However, several barriers to generate a neutralizing response against this region make MPER vaccine design considerably challenging^14^. Structurally, MPER peptide conformation is highly influenced by lipids and seems to be embedded into the viral membrane^29^,^30^, which is unusually rich in cholesterol (CHOL) and sphingomyelin (SM)^31^. Accordingly, anti-MPER bNAbs show cross-reactivity against lipids that seem to be essential for their neutralizing capacity^32^,^33^,^34^,^35^.

In order to dissect how lipids may modulate the immunogenicity of the MPER region, we generated a collection of proteoliposomes containing a gp41-derived miniprotein, previously shown to overexpose the MPER region^36^. To overcome potential limitation in MHC-II presentation after antigen processing in C57 BL/6 mice and to increase immunogenicity, the original gp41-Min protein was engrafted in the C-terminal moiety with a tetanus toxoid (TT) promiscuous T-helper epitope^37^,^38^. Furthermore, liposomes included several structural lipids overrepresented in the viral membrane (CHOL and SM)^31^ and lipids that may influence immune responses by promoting selective capture by antigen presenting cells (APC). In this regard, we tested phosphatidylserine (PS) that binds to different receptors on the surface of phagocytic cells^39^,^40^, and the ganglioside GM3, which binds to CD169 (SIGLEC-1) on the membrane of subcapsular sinus macrophages^41^,^42^, a highly specialized antigen presenting cell population that play a pivotal role during the induction of the humoral response^43^. Moreover, monophosphoryl lipid A (MPLA), a ligand of TLR4, was included as molecular adjuvant^44^.

By using this proteoliposome collection, we systematically evaluated the individual contribution of lipid components over MPER immunogenicity in mice, obtaining more than 2-log difference in serum antibody titers. We also mapped the responses elicited within the sequence of our immunogen. Remarkably, we found that proteoliposome presentation shifted the response from the TT peptide to the gp41 moiety of the immunogen. Moreover, regardless of the lipid composition used, the response targeted an epitope overlapping the 2F5-binding motif, revealing the existence of a gp41 immunodominant region that cannot be bypassed by any lipid mixture.

## Results

### Antigen design, production and characterization

**Figure 1:**
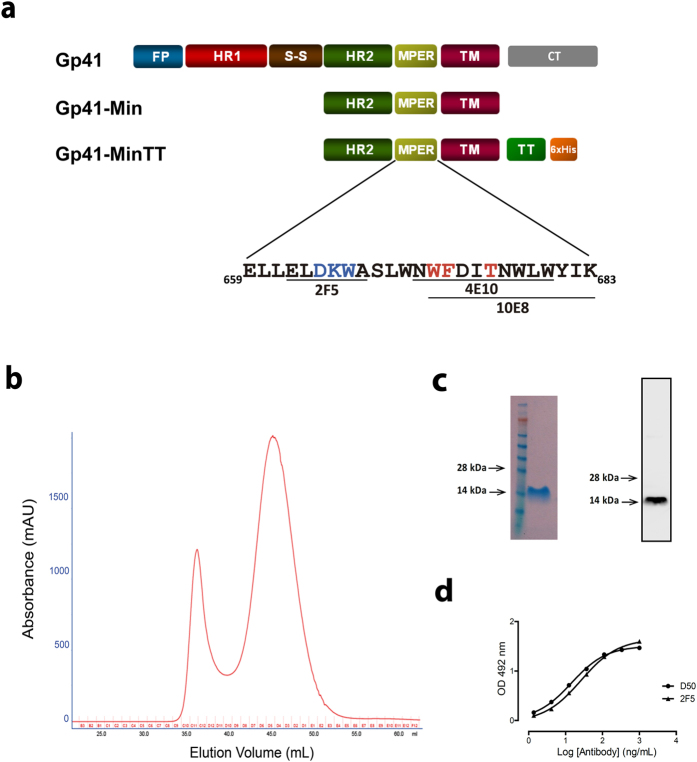
Gp41-MinTT expression and purification. (**a**) Schematic representation of gp41, gp41-Min and gp41-MinTT proteins is shown. FP, fusion peptide (blue); HR1, N-terminal heptad repeat (red); S-S, disulfide loop (brown); HR2, C-terminal heptad repeat (green); MPER, membrane proximal external region (yellow); TM, transmembrane domain (purple); CT, cytoplasmic tail (gray); TT, tetanus toxoid epitope (light green); 6xHis, 6-histidine tag (orange). MPER-spanning sequence (residues 659–683, HXB2 numbering) is depicted. MPER sequences containing the 2F5, 4E10 and 10E8 epitopes are underlined. 2F5 neutralizing core and residues equally recognized by 4E10 and 10E8 are highlighted in blue and red respectively. (**b**) Gel filtration chromatography. Elution profile of the latter purification step is shown. (**c**) A highly pure 15KDa protein was recovered and concentrated from central fractions of the largest peak shown in panel B (44–49 mL fractions), as confirmed by SDS-PAGE and coomassie staining (left) and by Western blot using the 2F5 antibody (right). Molecular markers are indicated. (**d**) Antigenicity of purified gp41-MinTT protein determined by ELISA using serial dilutions of D50 (anti-HR2) and 2F5 (anti-MPER) antibodies.

From a recently described collection of gp41-based miniproteins^36^, we selected a minimal gp41-based construction that greatly increased the MPER exposure, designed as gp41-Min. This protein contains the C-terminal heptad repeat (HR2), the MPER and the transmembrane (TM) domains of gp41. In order to overcome potential limitations in MHC-II presentation of the processed antigen^37^, a TT promiscuous T-helper epitope _830_QYIKANSKFIGITEL_844_^38^ was fused in frame with the TM domain. A six-histidine tag was added at the C-terminal end to allow protein purification. This construct, designed as gp41-MinTT (Fig. 1a), was produced in *E. coli.* After metal affinity, gel filtration chromatography yielded a highly pure 15 kDa gp41-MinTT recombinant protein peak (Fig. 1b). The integrity of the protein was confirmed by SDS-PAGE/coomassie staining, Western blot and ELISA using the specific antibodies D50 (HR2) and 2F5 (MPER) (Fig. 1c,d).

### Proteoliposome production and characterization

**Figure 2:**
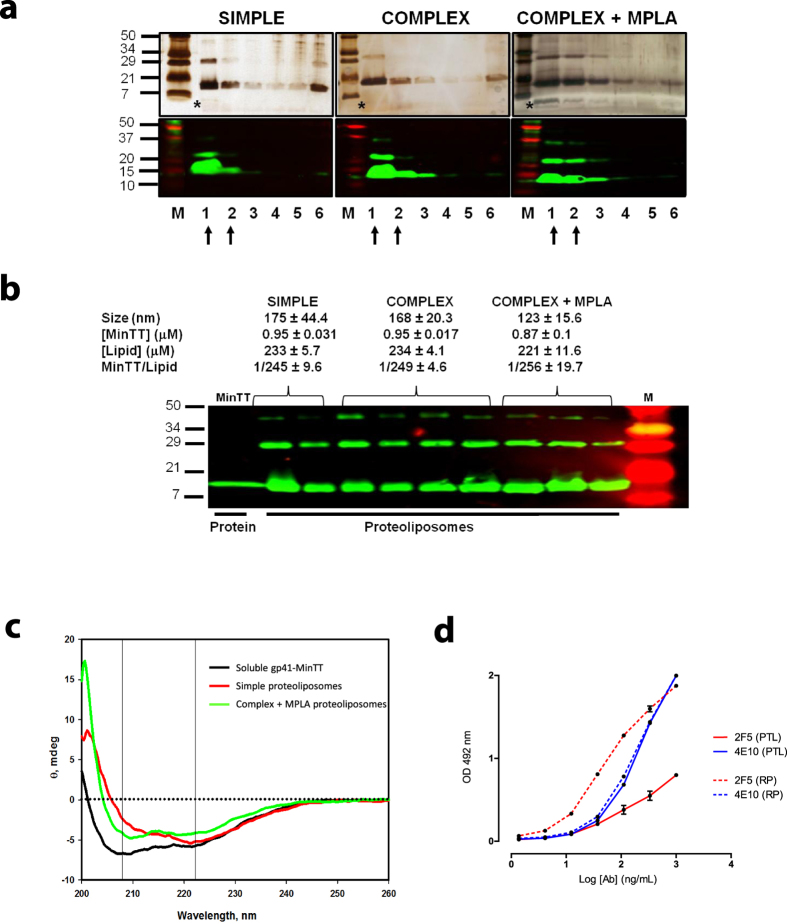
Proteoliposomes characterization. (**a**) Silver stained gels (top) and Western blots (bottom) of each sucrose gradient fractions corresponding to representative proteoliposomes of different composition (simple, complex and complex + MPLA). After proteoliposomes floatation, a sample of each recovered fraction was used for silver staining and Western blot analysis. Proteoliposomes could be detected in the first two fractions (arrows) by the signals of the protein and lipids (asterisks). M: Molecular Weight Marker and numbering 1 to 6 corresponds to the collected fractions from the top to the bottom of the ultracentrifuge tube. (**b**) Mean values and standard deviations of vesicle size, quantified protein and lipid amounts of each proteoliposome group are indicated. Ranges of protein/lipid ratios are also shown. Western blot analysis of the selected proteoliposome fractions for immunization is shown below. Purified gp41-MinTT protein was used as reference (left lane). (**c**) Secondary structure determination by circular dichroism. Near UV Spectra of gp41-MinTT in solution (black), simple (red) and complex + MPLA (green) proteoliposome formulations. Data are representative of two independent preparations. (**d**) Comparison of the antigenicity of gp41-MinTT-containing proteoliposomes (PTL, solid lines) and gp41-MinTT recombinant protein (RP, dotted lines) by ELISA using 2F5 (red) and 4E10 (blue) antibodies. Graph shows specific signal (OD 492 nm) of serial dilutions of monoclonal antibodies against both antigens captured by D50 antibody. Samples were assayed in duplicate and values are expressed as the mean and the interquartile range. Data are representative of two independent experiments.

Since membrane environment influences the conformation of the MPER^29^, we generated gp41-MinTT-based proteoliposomes using POPC and lipids overrepresented in the viral membrane-like environment, such as CHOL or SM^31^,^45^,^46^. The molar ratios chosen mimic viral membrane composition and rigidity^46^. The proteoliposomes used in this study were classified as complex or simple according to the presence or absence of CHOL and SM, respectively. In addition, we included lipids that bind to several receptors on the surface of antigen presenting cells (APC), such as the GM3 ganglioside^41^ and PS^47^, to seek whether the route of presentation may influence the immunogenicity of gp41-MinTT. We selected a GM3 molar ratio that induces optimal capture by dendritic cells^42^. The same amount of PS was used. Finally, we evaluated the effect of the TLR4 agonist MPLA over the effect of complex proteoliposomes. A summary of the composition of the proteoliposomes used in this study is specified in Table 1. Proteoliposomes were labeled with rhodamine, which was incorporated as DHPE-Rho into lipid mixtures. Proteoliposomes were purified by ultracentrifugation on a sucrose gradient. Since proteoliposomes have a lower density than the protein alone, they float up and are recovered from the first two fractions (Fig. 2a). Protein signal was detected by silver staining that also allowed visualization of fluorescent lipids (Fig. 2a). Fluorescence signal was measured by plate reader to quantify lipid content, while protein content was assessed by quantitative Western blot in a LiCoR system, which allows measuring the signal of each sample in the linear range. All proteoliposomes incorporated more than 85% of gp41-MinTT protein and showed an overall size distribution between 100–200 nm (Fig. 2b). The lipid amount ranged between 220–240 μM with a protein/lipid molar ratio always close to 1:250 (Fig. 2b). The effect of lyophilization and reconstitution on the protein content and size of proteoliposomes was routinely assessed by western blot and dynamic light scattering (DLS) respectively demonstrating no major differences before and after reconstitution (Supplemental Fig. 1). Consequently, all the proteoliposomes prepared were similar in size and lipid to protein mole ratio and, therefore, suitable for comparable immunization.

Interestingly, in the absence of lipids, gp41-MinTT protein showed a single monomeric band whereas in the presence of lipids, SDS and β-mercaptoethanol-resistant oligomeric bands appeared (Fig. 2b). Further characterization of the secondary structure of gp41-MinTT in solution and embedded in lipid mixtures (simple or complex + MPLA) was performed by circular dichroism (CD). Gp41-MinTT protein in solution showed predominantly an alpha-helix secondary structure with a positive absorption below 200 nm (data not shown) and two negative bands at 208 and 222 nm. Due to the complexity of proteoliposome samples, the spectra were recorded from 200 nm, since below this wavelength signal to noise ratio was too low. In the presence of lipids, the positive peak shifted to the red, and negative bands showed general loss of signal (Fig. 2c). This effect was more pronounced for the 208 nm band in simple proteoliposome preparations. The shift of the positive band, together with the Ө_222_/Ө_208_ ellipticity ratio >1, would support the existence of helix-helix interactions such as those described for heterodimeric coiled-coils^48^,^49^,^50^,^51^ and/or transmembrane helical bundles^52^. Seemingly, the protein in solution shows a Ө_222_/Ө_208_ ellipticity ratio <1, which would be consistent with the disruption of helix-helix interactions or monomeric alpha-helix^50^. Finally, we evaluated the antigenicity of MPER in ELISA assays comparing the binding of 2F5 and 4E10 antibodies to gp41-MinTT presented as a recombinant protein or exposed on complex proteoliposomes (Fig. 2d). The results showed that 4E10 equally recognized both preparations. In contrast, 2F5 showed a reduced binding to proteoliposomes, suggesting that the 2F5 epitope was differently exposed in both contexts. To further assess the orientation of the immunogen on proteoliposomes, the exposure of the C-terminal end of gp41-MinTT protein was measured by ELISA. The results showed that the C-terminal end was poorly exposed on the outer surface of proteoliposomes (Supplemental Fig. 2). Therefore, these data indicated that most of the immunogen was properly exposed. The gp41 moiety was displayed on the surface whereas the C-terminal end was oriented towards the lumen of proteoliposomes.

### Gp41-MinTT immunogenicity can be enhanced by modifying the lipid composition

**Figure 3:**
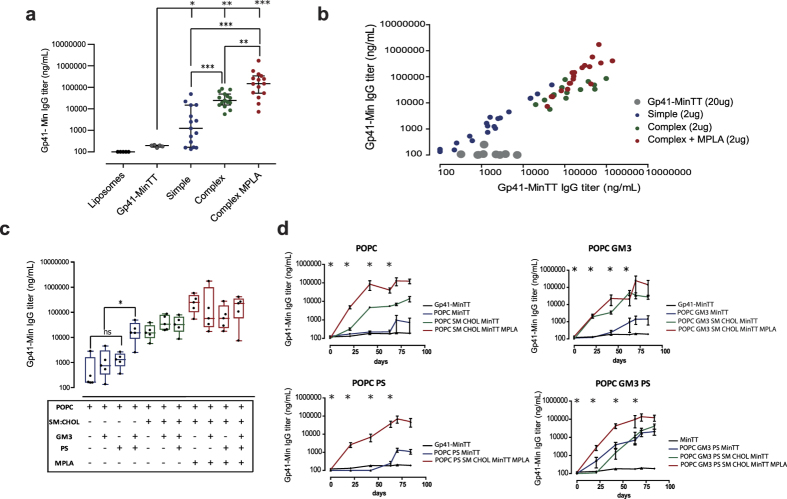
Effect of lipid composition on anti-gp41 IgG response. (**a**) Summary of anti-gp41-Min specific IgG titers, at sacrifice day, of C57 BL/6 mice immunized with gp41-MinTT proteoliposomes of simple (blue), complex (green) and complex incorporating MPLA (red) compositions. Data from animals immunized with control liposomes (black) and soluble gp41-MinTT protein (grey) are included. (**b**) Correlation of IgG titers, at sacrifice, against gp41-MinTT and gp41-Min antigens of mice immunized with gp41-MinTT as recombinant protein or formulated in proteoliposomes. (**c**) Influence of lipid mixtures over gp41-MinTT immunogenicity. Gp41-IgG titer at sacrifice day is shown. (**d**) Evolution of the gp41-Min IgG response of animals immunized with gp41-MinTT proteoliposomes based on POPC (upper left); POPC and GM3 (upper right); POPC and PS (down left); POPC combined with GM3 and PS (down right) in a simple, complex or complex with MPLA composition (blue, green and red lines respectively). Asterisks indicate immunization time points. In all panels, IgG titer is displayed as ng/mL referred to the D50 antibody, which was used as standard. Data show the median and interquartile range of at least two independent determinations. In panels (**a**) and (**c**), ***, ** and * denote p < 0.0001, p < 0.001 and p < 0.01 respectively. *ns*, not significant.

C57 BL/6 mice were immunized four times with 20 μg of proteoliposomes (containing 2 μg of gp41-MinTT protein) or with 20 μg of soluble recombinant protein in a 12-weeks immunization regimen. Three weeks after the last inoculum, anti-gp41 IgG titers were determined by ELISA using the gp41-Min protein, lacking the TT epitope^36^. Despite the lower inoculum dose used, all proteoliposome-immunized animals showed higher titers than those immunized with soluble protein (Fig. 3a). A global comparison between animals immunized with simple and complex proteoliposome formulations showed at least 1-log anti-gp41 titer increase in the complex group, indicating the enhancing effect of CHOL/SM addition (Fig. 3a). When we evaluated the adjuvant activity of MPLA, we found an additional 1-log titer increase, highlighting the potential benefits of this TLR agonist on anti-gp41 responses (Fig. 3a). Moreover, the combined analysis of antibody titers against recombinant gp41-MinTT and the original gp41-Min proteins showed that mice immunized with recombinant gp41-MinTT presented gp41-MinTT binding in the absence of gp41-Min titers, indicating that the response was exclusively focused against the immunogenic TT epitope (Fig. 3b). In contrast, a strongly correlated response against both proteins (r = 0.8373, p < 0.0001) was found for proteoliposomal preparations, indicating that the humoral response generated by those formulations was mostly directed against the gp41 domain. These findings demonstrate that the humoral response induced by gp41-MinTT immunogen can be dramatically modulated by the addition of lipids, which not only increase the magnitude of antibody titers but also guide the response towards the gp41 domain.

We next evaluated the effect of individual lipid components over gp41-MinTT immunogenicity (Fig. 3c,d). Although a slight but not significant effect was observed in terms of immunogenicity when GM3 or PS were individually incorporated into simple compositions (Fig. 3c), we detected faster kinetics of anti-gp41 antibodies in GM3-containing liposomes (Fig. 3d). In spite of this, the individual contribution of GM3 or PS to the global response was not statistically significant in any of the conditions tested (Supplemental Fig. 3). However, a great increase in antibody titer was detected when both components were formulated together in the context of simple POPC formulations (Fig. 3c). This synergistic effect was not additive to the enhanced effect caused by either CHOL-SM or MPLA, since it was lost in complex formulations (Fig. 3c). Remarkably, we observed that kinetics of the antibody response were faster in those animals immunized with complex proteoliposomes as well as for those receiving GM3 and PS in combination (Fig. 3d).

### Immunodominant response is focused on the N-terminal MPER region

**Figure 4:**
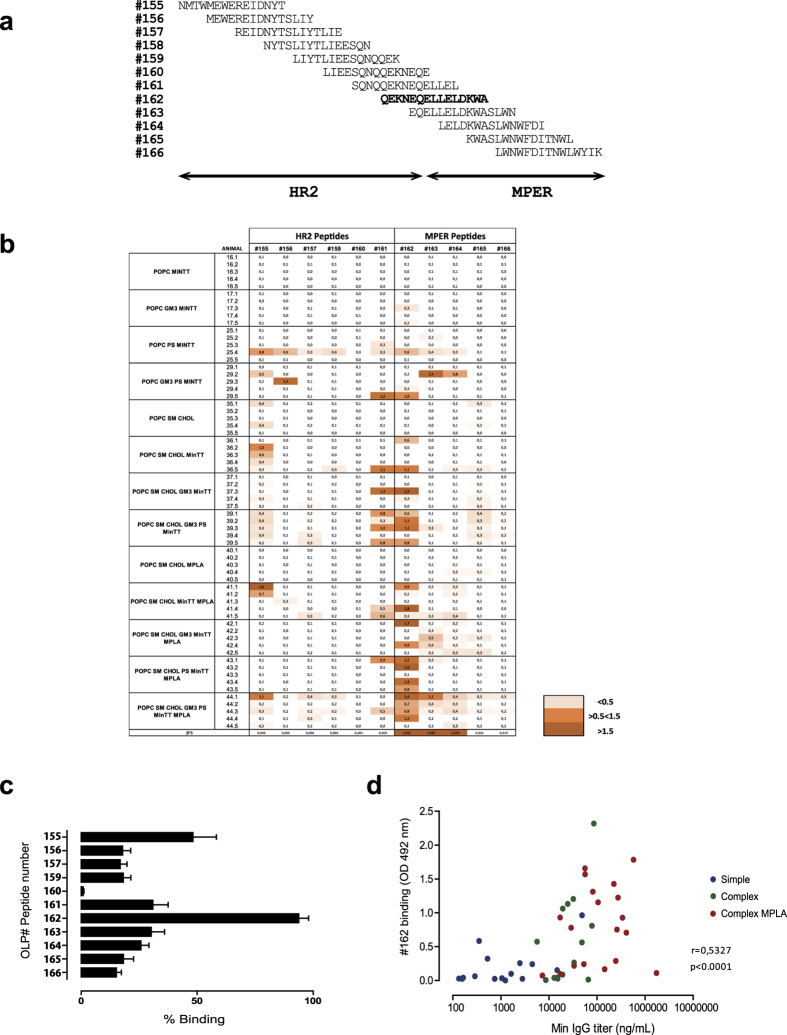
Mapping of humoral response against gp41-overlapping peptides. (**a**) 15-mer overlapping peptide collection covering the HR2 (OLP peptides #155–161) and the MPER (OLP peptides #162–166) used for mapping. (**b**) OD values of the indicated immunized mice sera (60-fold dilution) against OLP peptides in ELISA. 2F5 binding is also indicated. Color code is shown. (**c**) For each animal the peptide yielding the strongest signal was assigned 100%. Bar diagram shows the percentage of signal of each peptide in all animals (mean+/− SD), highlighting the immunodominance of the #162 peptide sequence. (**d**) Spearman’s correlation of #162 binding signals and gp41-Min titer of immunized mice sera. Data are representative of two independent experiments. Spearman’s correlation coefficient and p value are indicated.

In order to explore the gp41 regions targeted in immunized animals, we assayed sera against a collection of 15-mer overlapping peptides covering the HR2 and the MPER of gp41 (Fig. 4a). The results, summarized in Fig. 4b, showed that the global response was mostly directed against a peptide, designed as #162 (residues 653–667), that overlaps between HR2 and MPER and includes the 2F5 binding domain (Fig. 4c). This major response correlated with the global anti-gp41-Min titer (Fig. 4d), indicating that humoral responses against this region are especially favored by gp41-MinTT immunogen formulated within a membrane environment. Furthermore, complex formulations or addition of MPLA did not modify the specificity of the response that remained focused almost exclusively on the #162-covering region (Fig. 4b). We found hence that immunization with gp41-MinTT-based proteoliposomes generated an immunodominant response overlapping the N-terminal part of the MPER region, which contains the 2F5 binding domain.

**Figure 5:**
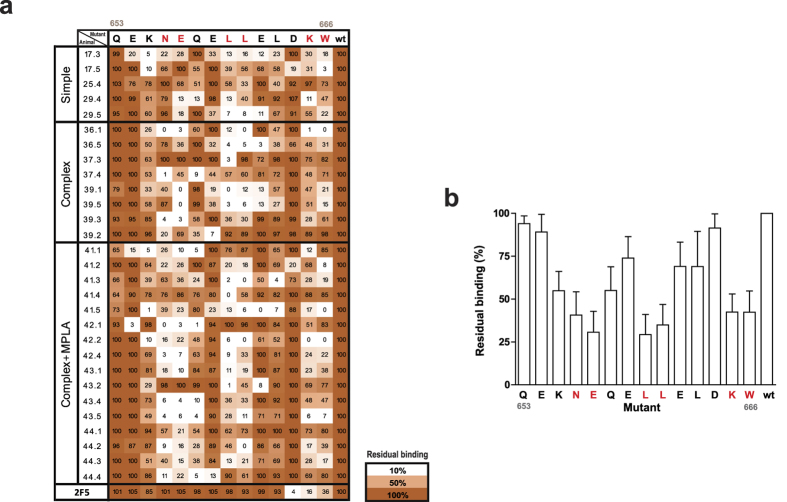
Alanine-scanning analysis of the immunodominance against #OLP-162. (**a**) 29 mice sera displaying the highest signals against OLP#162 peptide were tested for binding against a collection of OLP#162-alanine mutants. Values indicate the binding percentage relative to the wild type peptide signal. 2F5 profile is also included. Color code is indicated. (**b**) Percentage of residual binding (mean+/− SD) for each alanine mutant peptide is shown. Immunodominant residues (<50% residual binding) are highlighted in red in both panels. HXB2 numbering of Q_653_ and W_666_ residues is indicated in grey.

To gain insights into the features of elicited antibodies, we analyzed the relevant residues for #162 binding. We selected 29 serum samples displaying the highest #162 binding signals, and we assayed those sera against a collection of peptides in which each position of the #162 sequence was substituted by an alanine residue (Fig. 5a). On average, we found that 6 residues (N656, E657, L660, L661, K665 and W666) were especially relevant for #162 binding (Fig. 5b). Importantly, four out of six of these residues are located within the MPER region and two of them (K665 and W666) correspond to the 2F5 neutralizing core (_664_DKW_666_)^53^. In contrast, only a reduced number of sera recognized the D664 residue within the 2F5 epitope. Furthermore, modest neutralization activity was observed against NL4–3 and BaL subtype B viruses (Supplemental Fig. 4), which correlated with binding to #165 and MPER peptides but not with reactivity against the #162 peptide (Supplemental Fig. 5). In addition, no differences in the binding pattern to #162 peptide residues between neutralizing and non-neutralizing sera were observed (data not shown). Therefore, the lack of reactivity against the C-terminal moiety of the MPER may have precluded the elicitation of more potent neutralizing responses.

### Modeling of the immunodominant epitope in proteoliposomes

**Figure 6:**
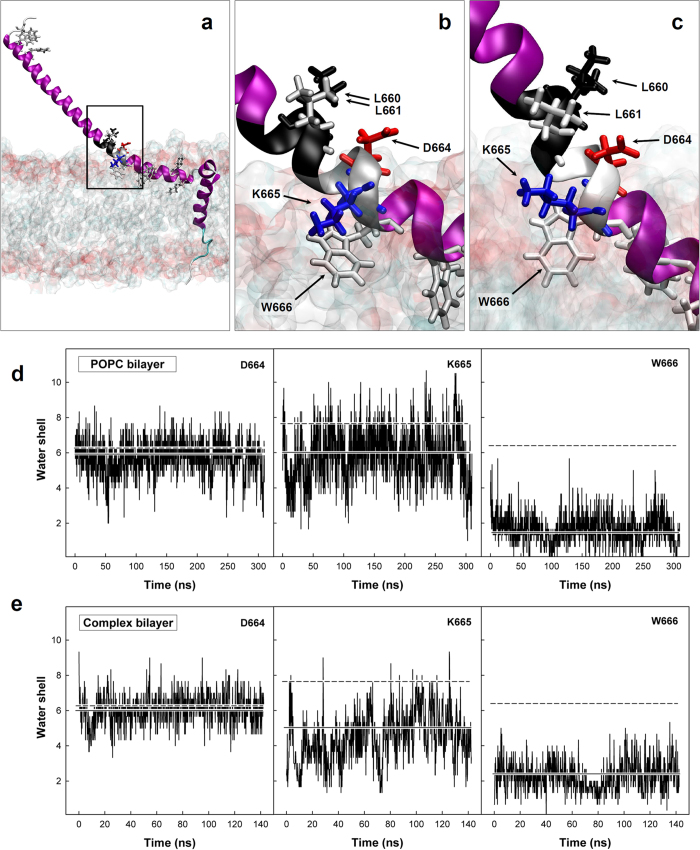
*In silico* structural model of gp41-Min. (**a**) Structural model embedded in a POPC bilayer. (**b,c**) Detail of the helix turns containing relevant residues for #162 binding in POPC (**b**) and complex (**c**) bilayers. Gp41-Min protein is shown in a ribbon representation. The helix turn containing L660 and L661 is shown in black and the turn containing W666 is shown in grey. In addition, all tryptophans present in gp41-Min and some residues specifically named in the text are labeled with arrows and represented as sticks. Lipid bilayers are represented as solvent accessible surface, truncated along the main protein axis to allow its visualization. (**d,e**) Number of water molecules within a sphere of 3 Å around lateral chains of residues D664 (left), K665 (middle) and W666 (right) in the gp41-Min model embedded in a POPC (**d**) and complex (**e**) bilayers throughout the molecular dynamics. Mean values are represented by a continuous horizontal line. The respective mean values obtained from the molecular dynamics performed in the absence of the lipid bilayer (water-only solvated model) are also shown for comparison (discontinuous line).

The lack of reactivity against the D664 residue and the immunodominance of peptide #162 were intriguing. In order to understand this reactivity, we generated an *in silico* model of the extracellular and TM sequences of gp41-Min protein embedded in a POPC or in a complex lipid bilayer. Molecular dynamics in POPC showed that the 6 relevant residues for #162 binding belong to two different turns (turn A, segment _659_ELLE_662_; turn B, segment _663_LDKW_666_) of the MPER helix that are located on the membrane interface (Fig. 6a,b). The turn B, strongly interacts with the lipid bilayer throughout the dynamics (W666 with the hydrophobic environment of the bilayer and K665 with phospholipid polar heads; total non-bond interaction energy of −55.2 kcal/mol), similar to the following two turns (turn C, residues A667 to W670: −38.6 kcal/mol; turn D, residues N671 to D674: −38.2 kcal/mol). On the contrary, the turn A containing N656, E657, L660 and L661, shows a much weaker interaction with the viral membrane (+5.4 kcal/mol). Similar results were found for the gp41-Min model embedded in a complex bilayer (turn A: +12.6 kcal/mol; turn B: −85.7 kcal/mol; turn C: −130.0 kcal/mol and turn D: −44.7 kcal/mol, Fig. 6c). Considering the immunodominance of W666 and K665 residues and the proximity of the polar D664 (Fig. 6a,c), we explored whether hydration patterns could help to identify hot spots for strong protein-protein interactions. Polar residues, as K665 and D664, are extremely solvatable in water solution, making weaker interactions with other polar solvents than when they are in a water excluded patch^54^. We compared the water shell around D664, K665 and W666 for the membrane embedded protein models and the water-only solvated model. We observed that W666 and K665 showed a lower level of hydration in both lipid bilayers when compared to soluble forms (Fig. 6d,e), while hydration of D664 was identical in both models, suggesting that extraction of W666 from the membrane will modify the hydration shell for K665 in a much larger extent than D664. In addition, the proximity of W666 prevents water molecules to fully solvate the lysine residue, thus, offering a much favorable propensity to create strong charge-charge interactions with other proteins. Overall, this behavior may explain the observed selectivity of antibody binding to K665 and the similar immunodominance observed for the different lipid compositions.

## Discussion

Anti-MPER bNAbs share a common neutralization mechanism in which interactions of CDRH3 hydrophobic residues with membrane lipids seem to be essential^32^,^55^,^56^,^57^,^58^,^59^. Additionally, MPER structure is influenced by membrane lipid composition^29^,^30^. Therefore, it is widely assumed that the generation of a robust anti-MPER response should require its presentation within a membrane environment to properly present neutralizing determinants and to implement lipid cross-reactivity^55^,^59^,^60^,^61^,^62^.

In this study, we evaluated to what extend lipids may modulate the MPER immunogenicity. Accordingly, we selected an immunogen containing the HR2, the MPER and the TM domain of gp41 resulting in an increased MPER exposure compared with the whole gp41 protein^36^. Additionally, we incorporated a promiscuous T-cell TT epitope to harness the MHC-II antigen presentation pathway that has been shown as a major limiting factor in MPER responses in murine models^37^. Likewise, in a cohort of untreated HIV-1 infected viremic controllers it has been recently shown an association between gp41-specific CD4 T cell responses with the magnitude and breadth of anti-Env neutralizing antibodies^63^. We incorporated gp41-MinTT protein into liposomes of diverse composition. Starting from POPC-based liposomes, we increased the complexity by adding (i) CHOL/SM, to mimic the viral membrane^31^; (ii) GM3 and PS, to modulate delivery to specialized APCs (such as the CD169+ subcapsular sinus macrophages)^43^,^47^,^64^,^65^,^66^ and (iii) the monophosphoryl Lipid A, a TLR4 agonist approved for human use, as a molecular adjuvant^44^.

Our data show that animals immunized with the recombinant protein gp41-MinTT developed a weaker humoral response than those immunized with 10-fold lower inoculum dose of the same protein formulated as proteoliposomes. Interestingly, humoral response elicited by soluble protein was focused on the TT peptide, while proteoliposomes guided the humoral response towards the gp41 extra-cellular portion of the recombinant protein, albeit the TT epitope was also exposed (data not shown). Secondary structure determination of the protein embedded in liposomes suggests a mostly alpha-helix structure, with some level of protein-protein interaction, absent in the soluble form. This is consistent with the oligomeric forms detected by SDS-PAGE analysis. Nevertheless, CD data only provides information on the overall secondary structure of the protein, without specific epitope structure. Therefore, differences in oligomerization status in soluble and proteoliposomal preparations, and changes in MPER exposure induced by lipids mimicking a native conformation, are probably reasons for enhanced immunogenicity of the gp41 moiety. Furthermore, we observed modest contributions of individual GM3 and PS incorporation into POPC proteoliposomes, although synergistic activity was noticed in this context, probably due to multivalent presentation of both ligands through different pathways. In contrast, incorporation of CHOL and SM significantly increased the gp41-MinTT immunogenicity compared to the simplest POPC proteoliposomes. This immunogenicity boost supports the notion that certain lipids may modulate the MPER structure and/or accessibility by modifying its membrane insertion index^29^,^67^ or membrane fluidity, accounting for a higher antibody response^68^,^69^. The membrane of HIV-1 virions shows an unusual high content of sphingolipids and CHOL^31^, which seems to be essential for MPER-induced membrane fusion^70^,^71^. Furthermore, the C-terminal end of the MPER contains the LWYIK “Cholesterol Recognition Amino acid Consensus” (CRAC) motif by which MPER-CHOL interactions would be supported^72^. Despite this, molecular dynamics showed similar exposure of the immunodominant domain on the surface of simple and complex membrane. Consistently, no changes in targeted epitopes were observed among simple or complex proteoliposomes; thus, we cannot exclude that distinct liposome composition might modulate other membrane features (such as membrane structure) that may impact on the stability of membrane-dependent structures, antigen uptake by draining lymph nodes and the interaction with antigen presenting cells^47^. Finally, we have confirmed that addition of the TLR4 agonist MPLA, as a molecular adjuvant, considerably enhanced the immunogenicity of the immunogen. Therefore, the use of CHOL and SM-containing liposomes in combination with MPLA constitutes an excellent carrier system for MPER-based immunogens. The applicability of these carriers to other membrane-dependent antigens will be also worth exploring.

A deeper analysis showed that the response of immunized animals was focused on peptide #162, which overlapped the HR2 and the MPER regions including the 2F5 binding domain. This peptide is placed at the interface between the lipid-embedded domain of the MPER (C-terminal) and the solvent-exposed HR2 region. The immunodominant epitope identified in this peptide included two of the 2F5 neutralizing core residues, the K665 and W666, the latter one being essential for viral fusion^23^,^24^. The immunodominance of this region and the lack of response against the C-terminal domain of the MPER may be involved in the modest neutralizing activity observed in sera from immunized animals. This observation is surprising since, the C-terminal domain of the MPER seems to be properly exposed, as denoted by the similar binding of 4E10 to soluble and proteoliposomal preparations of gp41-MinTT protein. In addition, this immunodominance did not depend on the proteoliposome composition either, since it was detected regardless of the lipid composition used. Thus, breaking the immunodominance of peptide #162 might be required to extend the response towards the C-terminal moiety of the MPER and increase the chance to develop a neutralizing response. Among the different strategies to widen the response, the influence of the TM domain should be evaluated. Although the TM domain anchors the MPER to the liposome avoiding early antigen dissociation^69^, it may shift the response from the C-terminal to the N-terminal MPER helix^69^. This observation is particularly intriguing in the context of currently available structural data on the MPER-TM transition residues. Our model showed that the MPER displayed a mostly alpha-helix conformation, as expected from CD data and previously published MPER structures^73^. However, it shows some level of disorganization in the MPER-TM joining region due to the tendency of R696 to interact with the polar membrane surface. This structure contrasts with previously reported rigid alpha-helix structures of the MPER-TM joining region^74^. Finally, the inclusion of a native-like trimeric transmembrane domain, that may better control for the extent of immunogen oligomerization, should also be considered as it might favor better interactions with anti-MPER bNAbs^75^. Therefore, understanding the role of TM in MPER immunogenicity is probably a key issue to develop new MPER-based immunogens.

Despite a close specificity by the 2F5 binding motif, sera from immunized animals showed a modest neutralizing activity. Similarly, an study in rhesus macaques by Dennison *et al*. showed that antibodies that recognized the 2F5 neutralizing core epitope (_664_DKW_666_), with a preferential binding for the gp41 fusion intermediate, lacked neutralizing activity^62^. Moreover, llama immunized with a gp41 mini protein generated a polyclonal humoral response that failed to neutralize HIV-1. However, the later authors isolated a single chain antibody (VHH) that binds partially to the 2F5 epitope, but lacked binding to W666^55^. This antibody became neutralizing when was made bivalent and displayed a hydrophobic CDRH3 tip which was essential for neutralization, a common feature of many other anti-MPER bNAbs^55^,^56^,^58^. In our case, the low neutralizing activity detected in immunized animals, could be also related to the lack of binding to peptide #163 and the observed reactivity against the HR2 region of gp41, that may limit the accessibility of antibodies to the target epitope either in the native envelope trimer or in the fusion intermediate structures, which are thought to be the main target of MPER bNAbs^32^,^76^. Our data support the notion that antibodies elicited by proteoliposome immunization extract W666 from the membrane, as it has been proposed for other anti-MPER antibodies^73^; although the response observed here lacked D664 reactivity. Structural information suggests that hydration shells of the different residues may determine the strength of charge-charge interactions and therefore the folding structures, the exposure and the reactivity of these key residues. However, other factors such as lipid-induced oligomerization may have impacted immunodominance of specific residues. Further structural analysis may provide insights for fine tuning of the hydrophobic/solvent interactions of these key residues that might impact final neutralization activity.

Overall, the results presented here show that lipid modulation of membrane-dependent antigens impacts immunogenicity and elicits potent antibody titers. We propose that CHOL and SM-containing liposomes in combination with molecular adjuvants like MPLA constitute excellent vaccine platforms for MPER immunogens. Combining these carriers with redesigned immunogens able to modify the exposure and immunogenicity of immunodominant residues will help to specifically elicit MPER-neutralizing responses.

## Materials and Methods

### Immunogen construction

Protein gp41-Min has been previously described^36^. A linker of four glycines and a TT promiscuous T-helper epitope _830_QYIKANSKFIGITEL_844_^38^ were added in frame at the end of the transmembrane domain of the gp41-Min. The construct was generated by sequential PCR using the gp41-Min plasmid as template and the following primers: sense primer: Nco I-MinS 5′ TTTGGCCATGGTTTGGAATAACAT 3′ and the following three antisense overlapping primers: Min-TTas1: 5′ GTTCGCTTTAATATACTGGCCACCGCCACCAGCTC-3′, Min-TTas2 5′ ATGCCAATAAATTTGCTGTTCGCTTTAATATACTG-3′ and Min-TTas3 5′ TTTAACTCGAGCAGTTCGGTAATGCCAATAAATTTGCTG-3. The final construct was cloned into a pET21d (+) plasmid (Novagen), in frame with the C-terminal 6xHis tag, using the NcoI and XhoI restriction enzymes (Fermentas).

### Recombinant gp41-MinTT production

Gp41-MinTT was produced in *E. coli* BL21 DE3 strain (Invitrogen). Cells were transformed with the plasmid pET-21-d+ Min-TT and cultured in LB medium supplemented with ampicillin. Cells were harvested by centrifugation and lysed with 50 mM Tris-HCl, 100 mM NaCl, (pH = 8) buffer supplemented with 2 mg/mL of lysozyme. Crude extract was then treated with 0.5% of Triton X-100 and inclusion bodies were collected after centrifugation. Inclusion bodies were solubilized using an urea-containing buffer (8 M urea, 20 mM Tris-HCl, 500 mM NaCl, 30 mM imidazole, pH = 8). Gp41-MinTT protein was purified by sepharose-Ni^2+^ affinity chromatography (GE Healthcare). An additional gel filtration purification step (Hiprep 16/60 Sephacryl S200 HR, GE Healthcare) was performed in the presence of 1% sodium dodecyl sulfate, 20 mM Tris-HCl buffer (pH = 7). Purified gp41-MinTT protein was dialyzed against PBS and concentrated with a 3 kDa Amicon Ultra-15 device (Milipore) to reduce SDS concentration to <0.1%. Purity was assessed by SDS-polyacrylamide gel electrophoresis (SDS-PAGE) and G-250 coomassie staining (Biorad).

### Proteoliposome production and characterization

Large unilamellar vesicles (LUV) were prepared following the extrusion method described before^77^. Briefly, lipids (Avanti Polar Lipids) were mixed in chloroform:methanol (2:1) ratio (v/v) and dried under a nitrogen stream. Traces of organic solvent were removed by vacuum pumping for 1–2 h. Subsequently, the dried lipid film was dispersed in 50 mM MES, 50 mM Tris, 1 mM EDTA (pH 7.3) and subjected to 10 freeze-thaw cycles prior to extruding 10 times through two stacked polycarbonate membranes with a 100 nm pore size (Nucleopore) using the Thermo-barrel extruder (Lipex extruder, Northern Lipids, Inc.). To make proteoliposome fraction visible, 1% of Rho-DHPE (Molecular Probes) was added in the organic phase during LUV preparation. Highly pure Gp41-MinTT and Liposomes were mixed in a 1:250 molar ratio, 1:10 (w/w). Octylglucoside (OG) (Sigma-Aldrich) was the detergent used for proteoliposome preparation. OG was removed by extensive dialysis using 14 kDa membranes (Sigma-Aldrich) in 50 mM MES, 50 mM Tris, 1 mM EDTA (pH 7.3) buffer, changing the buffer solution every 4–8 hours for two days. The sample to buffer ratio used during dialysis was 1:2000 ratio (v/v). Newly formed proteoliposomes were subsequently purified by floatation using ultracentrifugation for 210 min through a sucrose gradient (0–25–30% of sucrose) in a TLA 120.2 rotor at 100,000 rpm. Each sample was in 30% sucrose. After ultracentrifugation, six fractions were collected. The proteoliposomes were layered in the first two fractions as proved by the rhodamine fluorescence and the silver staining of each fraction. Once the proteoliposomes were purified, they were immediately freeze-dried in a FreeZone lyophilizer (Labconco). Proteoliposomes were characterized based on their vesicle size distribution using a Malvern Zeta-Sizer instrument (Malvern Instruments, Malvern, UK), and their lipid/protein quantification. For lipid quantification membrane labeled rhodamine fluorescence was measured in a BioTek Synergy HT plate reader. In all cases, Triton X-100 was added to avoid fluorophore quenching effect induced by the order degree of different lipid compositions. Protein quantification was achieved by a quantitative Western blot using anti-Histidine (Invitrogen) antibody. Detection was carried out on a LI-COR Odyssey system (LI-COR Biosciences) following manufacturer recommendations. All proteoliposomal preparations were aliquoted, freeze-dried and stored at −30 °C, and reconstituted shortly before use.

### Secondary Structure Determination

Circular Dichroism (CD) measurements were carried out on a thermally-controlled Jasco J-810 circular dichroism spectropolarimeter calibrated routinely with (1S)-(+)-10-camphorsulfonic acid, ammonium salt. The structure of the purified gp41-MinTT protein was measured in 50 mM Tris, 1 mM EDTA (pH 7.3) buffer at 1 μM concentration and proteoliposomes were prepared as previously described using 1:250 protein to lipid molar ratio in POPC (simple) and POPC:SM:CHOL:GM3:PS:MPLA (Complex + MPLA) lipid composition. After floatation, only the first fraction was used for CD measurements. Buffer or protein-free liposomes signals were subtracted to all spectra. Spectra were recorded between 200–260 nm in a 0.2 cm path-length quartz cell equilibrated at 20 °C. Data were taken with 1 nm band-width, 20 nm/min speed, and the results of 5 scans were averaged. The measurements were done with two independent preparations with similar results.

### Experimental Animal modeling and Immunization regimens

Seven-week old female C57 BL/6 mice were purchased from Harlan Interfauna Iberica (Spain). The animals were shipped under suitable conditions, with the corresponding certificate of health and origin. Upon arrival, mice were kept under controlled conditions in a P3 high security facility with sterile food and water “ad libitum”. All animal procedures were performed in accordance with relevant guidelines and regulations and approved by the Animal Care Committee of the Germans Trias i Pujol University Hospital and the Department of Environment of the Catalan Government. Mice (five per experimental group) were subcutaneously immunized four times at weeks 0, 3, 6 and 9 with proteoliposome preparations (2 μg gp41-MinTT protein: 20 μg lipids/inoculum), liposomes (20 μg/inoculum) or recombinant gp41-MinTT (20 μg protein/inoculum), reconstituted in PBS buffer. Mice were examined daily following a protocol that monitored weight loss, apparent good health (bristle hair and wounded skin) and behavior (signs of aggressiveness or isolation). Blood samples were collected from facial vein before each immunization point as well as at week 10. Mice were euthanized at week 12 with isoflurane (inhalation excess) in order to avoid any suffering, and total blood and spleen were collected. Serum was obtained by blood centrifugation at 5000 × g for 10 minutes.

### Peptide and protein antigens

15-mer overlapping peptides covering the gp41 HR2 and MPER regions were obtained from the NIH AIDS Reagent Program (HIV-1 Consensus B Env Peptide Set, cat# 9480). Alanine mutants of peptide #162 (QEKNEQELLELDKWA) and a full MPER peptide (EQELLELDKWASLWNWFNITNWLWYIKL) were purchased from Covalab. Recombinant gp41-Min protein was produced as described previously^36^.

### Western blot and ELISA assays

For Western blot, samples were loaded onto a NuPAGE^®^ Novex^®^ 4–12% Bis-Tris Gel (Life Technologies) and blotted using in an iBlot Gel Transfer Device (Life Technologies). After blocking (5% w/v skimmed milk PBS buffer +0.05% v/v Tween 20), membrane was incubated at 4 °C overnight with 2F5 (Polymun) antibody. Membrane was then washed in PBS and incubated with Horseradish Peroxidase (HRP)-conjugated-F(ab)_2_ Goat anti-human IgG Fc specific (Jackson Immunoresearch). Membrane was developed using an Enhanced Chemioluminiscent HRP substrate (Fisher Scientific). For ELISA, recombinant gp41-Min protein and peptides were prepared at 1 μg/mL and 10 μg/mL in PBS or carbonate/bicarbonate buffer (pH = 9.6) respectively to coat 96-well Maxisorp Nunc-immuno plates (Fisher Scientific, 50 μl/well). After blocking with 1% bovine serum albumin (Sigma-Aldrich), plates were incubated with 100 ul of previously diluted serum samples overnight at 4 °C. Plates were then washed and 100 μl of a (HRP)-conjugated F(ab)_2_ Goat anti-mouse IgG (Fc specific, Jackson Immunoresearch) were dispensed for one hour at room temperature. Plates were developed with 100 μl of O-Phenylenediamine dihydrochloride (OPD) substrate (Sigma-Aldrich). Optical density was measured at 492 nm for specific signal and at 620 nm for background. A different strategy was followed to characterize the antigenicity and orientation of gp41-MinTT within proteoliposomes. Briefly, ELISA plates were coated with 50 ng/well of anti-gp41 monoclonal antibody D50 (NIH AIDS Reagent Program, cat#11393)^78^ or 2F5 (Polymun) and, after blocking, complex proteoliposomes (POPC:SM:CHOL) containing gp41-MinTT (intact or solubilized by using 0.5% of Tween-20 detergent) or recombinant gp41-MinTT protein (30 ng of protein/well in both cases) were added and incubated 2 hours at room temperature. Then, plates were incubated sequentially with serial dilutions of 2F5, 4E10 or anti-6xHis (HIS.H8 ThermoFisher, cat# MA1-21315) monoclonal antibodies (overnight at 4 °C) and with (HRP)-conjugated F(ab)_2_ Goat anti-human IgG or F(ab)_2_ Goat anti-mouse IgG (Fc specific, Jackson Immunoresearch, 1 hour at room temperature). Plates were developed as described above. Proteoliposome ELISA experiments were performed in absence of Tween 20.

### Viruses and neutralization assays

Pseudotyped HIV-1 were generated by cotransfection of Env expression plasmids and the pSG3 vector as described elsewhere^79^. Virus neutralization by sera samples was tested by a standard TZM-bl based assay. Briefly, in a 96-well culture plate, 100ul of previously heat inactivated (56 °C, 30 minutes) and 100-fold diluted plasma samples were preincubated with 50 ul of pseudovirus stock (200 TCID_50_) at 37 °C for one hour. Then, 100 ul containing 10,000 TZM-bl luciferase-reporter target cells and dextran (10 μg/mL) (Sigma Aldrich) per well were added. Plates were cultured at 37 °C and 5% CO_2_ for 48 hours. 2F5, 4E10 and IgGb12 (Polymun Scientific), and anti-CD4 clone SK3 (BD Biociences) were used as controls. Luciferase substrate (Britelite Plus, Perkin-Elmer) was used for the read out.

### Structural modeling

Firstly, an *in silico* structural model of the extracellular and transmembrane segments of gp41-Min (including a few residues of the intracellular segment, 707 to 714, to stabilize the transmembrane domain) was constructed using Modeller 9v14^80^. The RCSB Protein Data Bank (PDB) structures 3VGX and 3H01 were used as templates for HR2 and MPER regions, while 2MG1 structure was used as template for the transmembrane domain. Alignment was done using 3D-Coffee^81^. The best of six models obtained from Modeller, as assessed by the DOPE (Discrete Optimized Protein Energy) method, was embedded in a POPC bilayer using the VMD^82^ membrane plugin, solvated with TIP3P water molecules (15 Å as minimal distance to the edge of the box). Additionally, Cl^−^ and Na^+^ ions were added up to a final concentration of 0.15 M. The final structure was minimized, thermalized, and then submitted to about 310 ns of molecular dynamics at 310 K, using the CHARMM36 force field, with a time step of 2 fs, a cut-off distance for non-bonded interactions of 10 Å and Particle-Mesh-Ewald for long-range electrostatic interactions. The same protein structure in the absence of lipids was also submitted to a shorter molecular dynamics (150 ns) for comparative analysis. Protein RMSD throughout the dynamics for the two models is shown in Supplemental Fig. 6. Dynamics were performed with NAMD^83^ at BSC supercomputing facilities (Barcelona): Marenostrum (Barcelona) and Finisterrae (Vigo). VMD was used for protein dynamics analysis. Secondly, the protein structure previously minimized and thermalized in a POPC membrane, was embedded in a bilayer mimicking the lipidic composition of complex proteoliposomes (POPC:SM:CHOL) using the CHARMM-GUI Membrane Builder^84^. Then, the system was truncated (to obtain a size similar to the POPC bilayer), solvated and ionized with VMD, and finally submitted to about 150 ns of molecular dynamics in the same conditions referred above.

## Additional Information

**How to cite this article**: Molinos-Albert, L. M. *et al*. Proteoliposomal formulations of an HIV-1 gp41-based miniprotein elicit a lipid-dependent immunodominant response overlapping the 2F5 binding motif. *Sci. Rep.* **7**, 40800; doi: 10.1038/srep40800 (2017).

**Publisher's note:** Springer Nature remains neutral with regard to jurisdictional claims in published maps and institutional affiliations.

## Supplementary Material

Supplementary Information

## Figures and Tables

**Table 1:** Gp41-MinTT-containing immunogens used in this study.

FORMULATION	LIPID COMPOSITION (molar ratio)	PROTEIN	DOSE/INOCULUM
Recombinant protein		gp41-MinTT	20 μg
Simple proteoliposomes	POPC (100)	gp41-MinTT	2 μg
	POPC:GM3 (95:5)	gp41-MinTT	2 μg
	POPC:PS (95:5)	gp41-MinTT	2 μg
	POPC:GM3:PS (90:5:5)	gp41-MinTT	2 μg
Complex proteoliposomes	POPC:SM:CHOL (30:25:45)	gp41-MinTT	2 μg
	POPC:SM:CHOL:GM3 (30:20:45:5)	gp41-MinTT	2 μg
	POPC:SM:CHOL:PS (25:25:45:5)	gp41-MinTT	n.d.*
	POPC:SM:CHOL:GM3:PS (25:20:45:5:5)	gp41-MinTT	2 μg
Complex proteoliposomes + MPLA	POPC:SM:CHOL:MPLA (29:25:45:1)	gp41-MinTT	2 μg
	POPC:SM:CHOL:GM3:MPLA (29:20:45:5:1)	gp41-MinTT	2 μg
	POPC:SM:CHOL:PS:MPLA (24:25:45:5:1)	gp41-MinTT	2 μg
	POPC:SM:CHOL:GM3:PS:MPLA (24:20:45:5:5:1)	gp41-MinTT	2 μg

Immunogens were categorized in recombinant protein (gp41-MinTT), simple (POPC), complex (POPC CHOL SM) and complex + MPLA (POPC CHOL SM MPLA) proteoliposomes. GM3, PS or both were added to each category. Percentages are referred to lipid components. Lipid mole ratios were used. POPC: 1-Palmitoyl-2-oleoylphosphatidylcholine; CHOL, cholesterol; SM, sphingomyelin; GM3, monosialodihexosylganglioside; PS, phosphatidylserine; MPLA, monophosphoryl lipid A.

*n.d.: not determined.
