# Supplementary material for: Proteoliposomal formulations of an HIV-1 gp41-based miniprotein elicit a lipid-dependent immunodominant response overlapping the 2F5 binding motif
Source: Sci Rep. 2017 Jan 13;7:40800. doi: 10.1038/srep40800 (PMC5234007; doi:10.1038/srep40800)
Supplement: Supplementary Information [file srep40800-s1.pdf]

Proteoliposomal formulations of an HIV-1 gp41-based miniprotein elicit a lipid-dependent immunodominant response overlapping the 2F5 binding motif

Luis M Molinos-Albert, Eneritz Bilbao, Luis Agulló, Silvia Marfil, Elisabet García, Maria Luisa Rodríguez de la Concepción, Nuria Izquierdo-Useros, Cristina Vilaplana, Jon A. Nieto-Garai, F.-Xabier Contreras, Martin Floor, Pere J Cardona, Javier Martinez-Picado, Bonaventura Clotet, Jordi Villà-Freixa, Maier Lorizate, Jorge Carrillo, Julià Blanco

SUPPLEMENTARY INFORMATION

SUPPLEMENTARY FIGURE 1

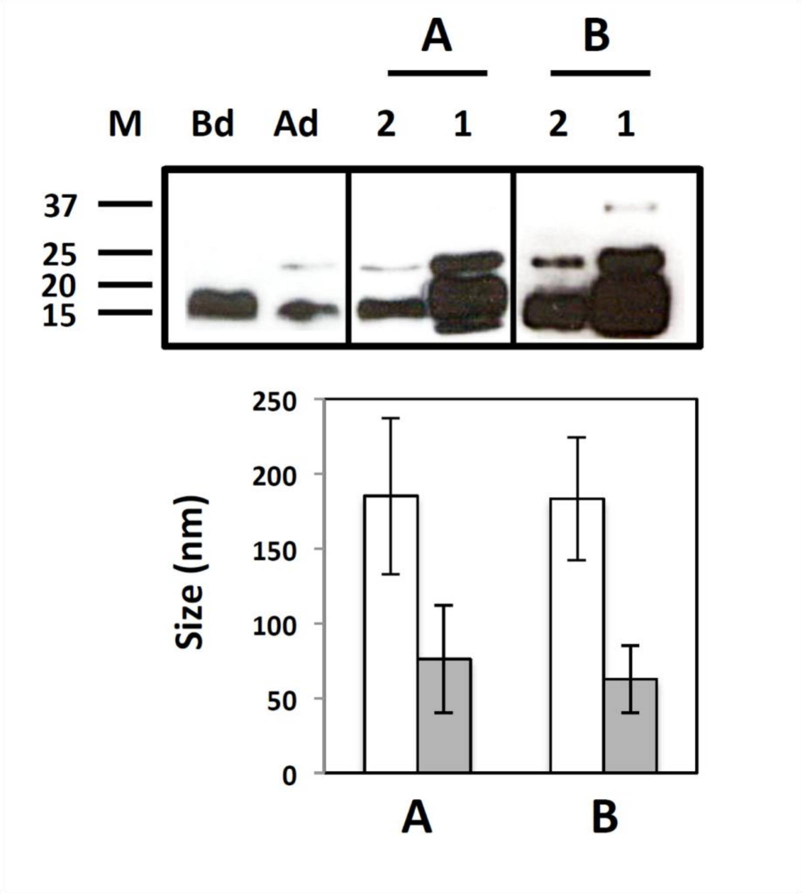

Supplementary figure 1. Western Blot analysis and size distribution of proteoliposomes before and after reconstitution in water. Western Blot analysis (up) of Gp41-MinTT, lipids and detergent mixture before dialysis (Bd), proteoliposome after dialysis (Ad) as well as 1 and 2 fractions of purified proteoliposomes before (A) and after (B) reconstitution. Below, size distribution of purified proteoliposomes before (A) and after (B) reconstitution. Z average in white and band width in grey. Proteoliposome mean polydispersion was 0,25 for A and 0,17 for B. Values are mean +/- SD of 6 independent preparations.

SUPPLEMENTARY FIGURE 2

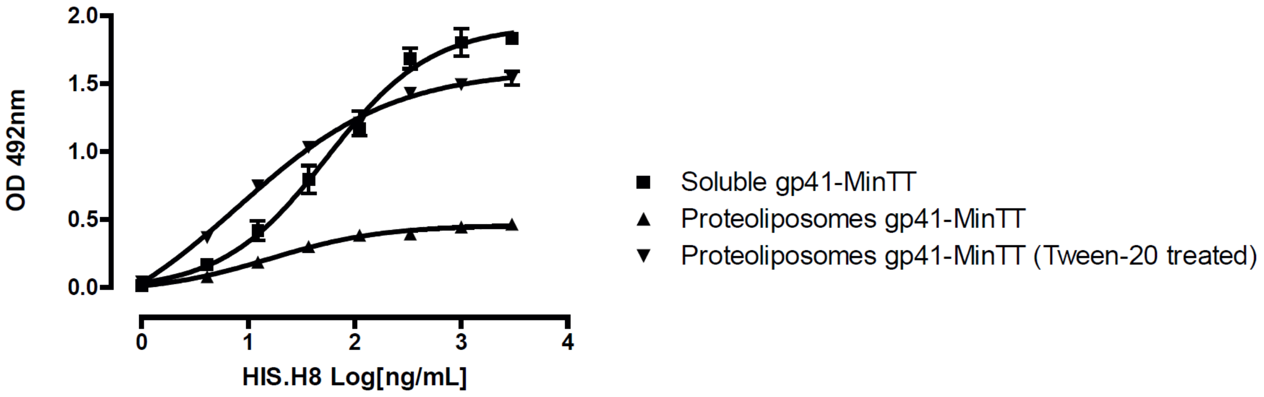

Supplementary figure 2. Characterization of gp41-MinTT protein orientation within proteoliposomes. Determination of histidine tag (6xHis) exposure within proteoliposomes. Representative sandwich ELISA experiment of gp41-MinTT as soluble protein (squares), proteoliposomes (face up triangles) and proteoliposomes treated with 0.5% of Tween-20 detergent (face down triangles). ELISA plates were coated with 100 µl of 2F5 antibody (1µg/mL) and after blocking, 100 µl/well of each sample containing 200 ng/mL of gp41-MinTT was added. Serial-diluted concentrations of mouse monoclonal antibody HIS.H8 (catalog number MA1-21315) was used to determine the level of exposure of the histidine tag. Read out was performed by an (HRP)-conjugated $F(ab)_2$ Goat anti-mouse IgG Fc specific antibody (Jackson Immunoresearch).

SUPPLEMENTARY FIGURE 3

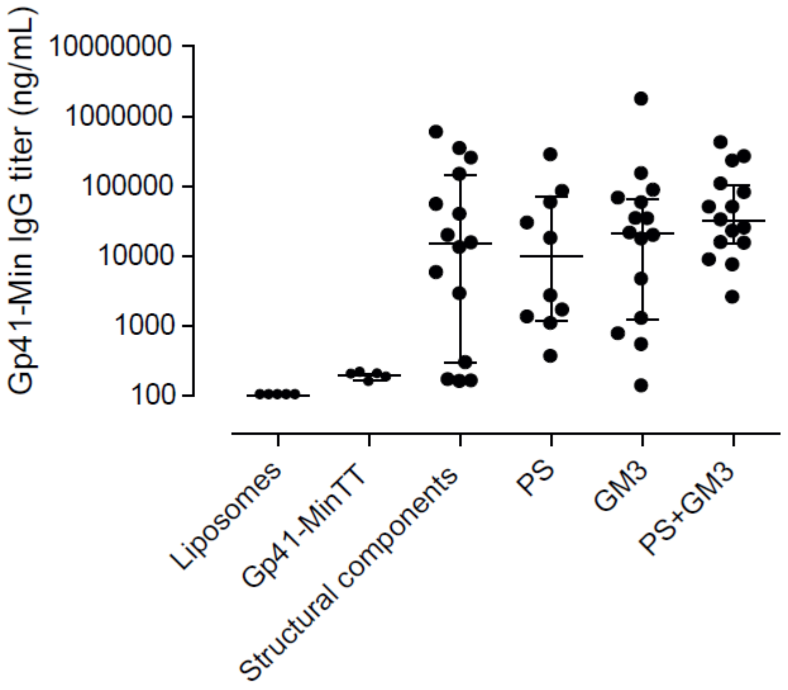

Supplementary figure 3. Individual contribution of PS and GM3 to gp41-MinTT immunogenicity. Gp41-Min specific IgG titers of immunized animals with liposomes containing only structural components (POPC with or without CHOL and SM) or adding GM3, PS or both are displayed.

SUPPLEMENTARY FIGURE 4

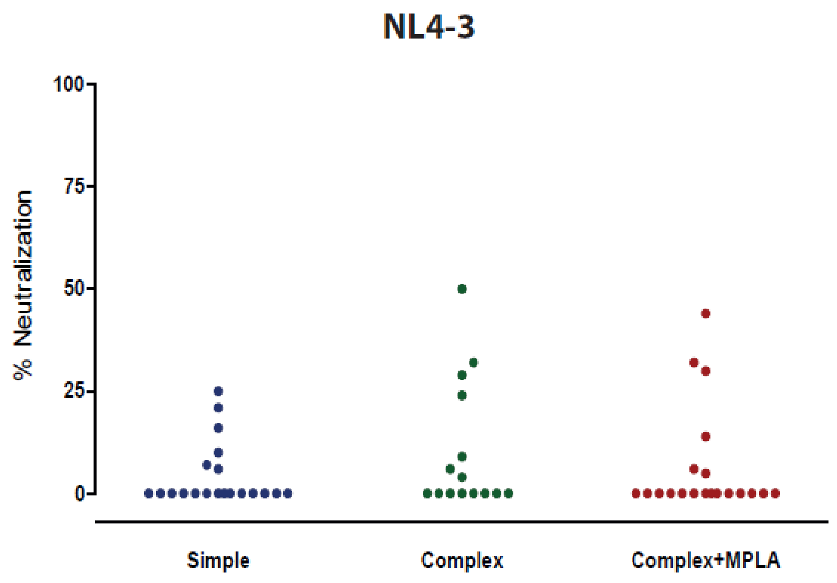

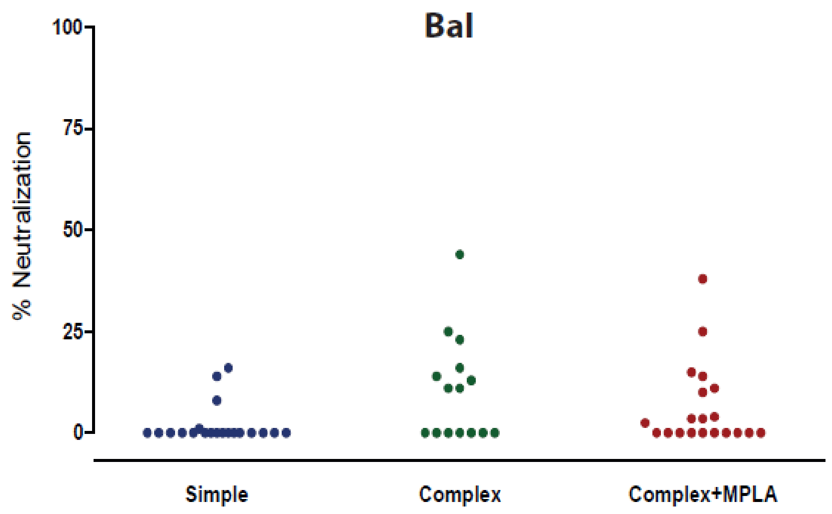

Supplementary figure 4. Neutralizing activity of sera from immunized animals. The ability of sera obtained at sacrifice was assessed in TZM-bl assays against the HIV-1 isolates NL4-3 and BaL at a single 1/100 dilution. The neutralizing activity is shown for the different groups of immunized animals

SUPPLEMENTARY FIGURE 5

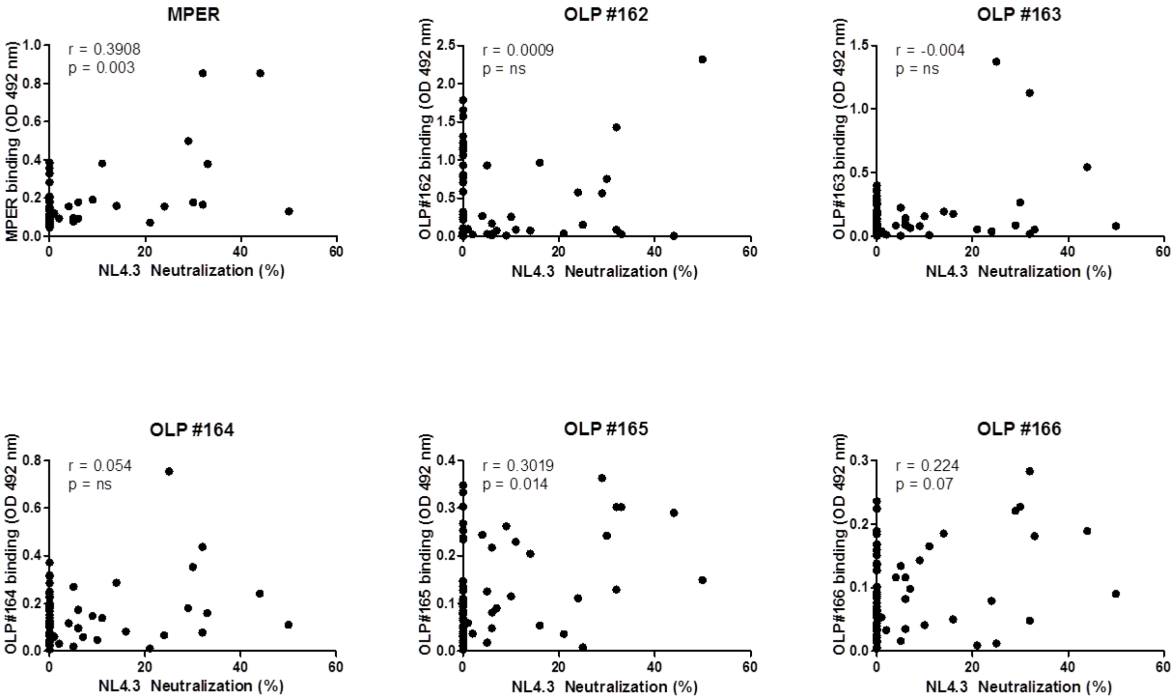

Supplementary figure 5. Correlation (Spearman test) of OLP peptide binding ELISA data with neutralization activity of immunized animal sera. Values were determined as described in Methods section of the manuscript

SUPPLEMENTARY FIGURE 6

A

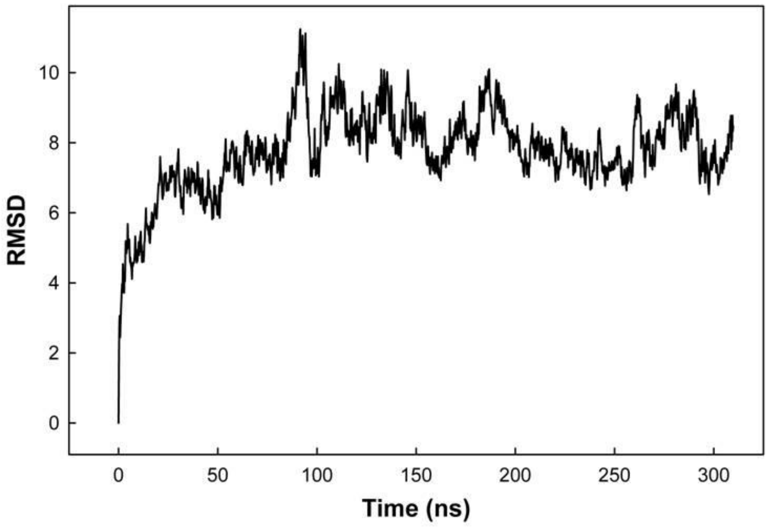

B

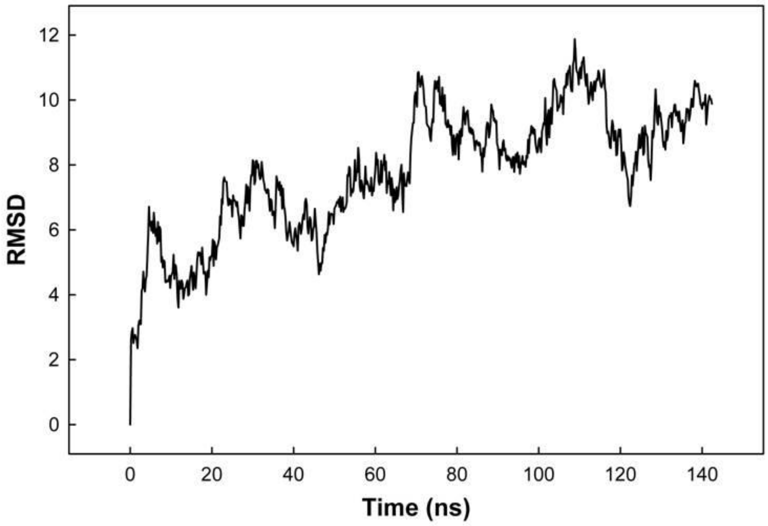

Supplementary figure 6. Root-mean-square deviation of atomic positions (RMSD) during molecular dynamics of the gp41-Min protein embebbed in a POPC bilayer (A) and complex bilayer (B).
